# Analysis of Shape Geometry and Roughness of Ti6Al4V Parts Fabricated by Nanosecond Laser Ablation

**DOI:** 10.3390/mi9070324

**Published:** 2018-06-27

**Authors:** Sabina Luisa Campanelli, Fulvio Lavecchia, Nicola Contuzzi, Gianluca Percoco

**Affiliations:** Dipartimento di Meccanica, Matematica e Management, Politecnico di Bari, Viale Japigia 182, Bari 70126, Italy; fulvio.lavecchia@poliba.it (F.L.); nicola.contuzzi@poliba.it (N.C.); gianluca.percoco@poliba.it (G.P.)

**Keywords:** laser milling, process parameters, 3D measurement, conoscopic holography, roughness

## Abstract

Laser milling is a micro-machining process that uses a laser beam as a tool to remove material through the layer-by-layer ablation mechanism. Generally in laser ablation, the quality of parts is reduced by melt accretions and thermal damage; therefore, this problem is reduced with shorter pulse duration, although ablation efficiency decreases as well. Thus, laser ablation in the nanosecond range still offers a good compromise between process quality and efficiency. Therefore, laser milling with nanosecond laser ablation requires an accurate study to reduce geometric defects induced by the process. The aim of this paper was to study the shape geometry and roughness of Ti6Al4V parts fabricated by laser milling using a nanosecond Nd:YAG laser source. The impact of the laser processing parameters on machining outcomes was studied in order to determine the optimized processing conditions for reducing geometrical defects and improving surface quality. In particular, the influence of average laser power, frequency, and scanning speed was investigated. The geometry of micro-parts was revealed using a 3D digitizing system, the Optimet Mini Conoscan 4000, which combines a non-contact, single-point measuring sensor based on conoscopic holography technology. The use of this measurement technology yielded complete information of the shape geometry and dimensions of the built parts. In addition, the roughness of manufactured surfaces was assessed to complete the analysis.

## 1. Introduction

Laser milling (LM) is an emergent process for micro-fabrication, consisting of material removal by a laser beam through layer-by-layer ablation.

LM shows several advantages compared to traditional manufacturing processes, such as the ability to work hard to machine materials such as ceramics, graphite, and cemented carbides, the totally absence of tool wear, surface finish, aspect ratio, dimensional accuracy, and minimum feature size [1,2,3,4].

Although several works can be found in literature on the effect of laser process parameters on quality of laser ablated parts, few of them focus on the analysis of the shape and dimensions of parts fabricated by laser milling.

Teixidor et al. [5] studied the effects of laser milling process parameters on the geometrical and surface quality of micro-channels fabricated on AISI H13 steel. They showed that the laser milling process displays geometrical defects when grooves or micro-features are manufactured. These defects exhibit the complexity of the laser milling process and clarify the need to find methods and models to determine the best conditions and predict results to improve the productivity and quality of the process.

Darwish et al. [6], investigated the fabrication of Inconel 718 micro-channels under dry and wet conditions, focusing on the analysis of the machined geometry in terms of width, depth, and wall shape angle. Geometries were measured with a scanning electron microscope (SEM) after cutting and polishing of samples.

Chen et al. [7] analyzed the influence of several micro channel structure laser carving features (channel aperture, channel lines, and channel distance) on the efficiency and FF of polycrystalline silicon solar cells.

Karazi et al. [8] developed models for the prediction of the width and depth dimensions of CO_2_ laser-formed micro-channels in glass. The width and depth dimensions of the micro-channels for each experiment were measured at three different locations along the produced channel. The measurement system used was an in-house built laser profilometer that had a 1.95 mm resolution in the x and y directions and a 0.5 mm resolution in the z-direction.

Schille et al. [9] studied performance of laser micro processing of metals in terms of ablation depth, wall-angle, and surface roughness. They used a confocal point sensor CF 4 and a tactile roughness device (DEKTAK 3030) for measurement of parts.

Bulushev et al. [10] measure micro-channels by a profilometer based on a confocal chromatic sensor and by a confocal microscope with higher lateral resolution, in order to measure the depth of micro channels using the first sensor and roughness and edge characteristics.

Rysava & Bruschi [11] compared micro-milled channels on an Electron Beam Melting (EBM) and Direct Metal Laser Sintering (DMLS) work pieces. The micro-machinability is evaluated in terms of burr formation, surface integrity (surface topography and surface defects), tool damage, and microstructure alterations. Scanning Electron and Confocal Microscopes are the measuring instruments employed. Generally, the election measuring instrument for 3D characterization is the Confocal Microscope.

On the other hand, the use of optical and confocal microscopes is limited when high slope walls should be measured. Conoscopic holography (CH), a non-contact interferometric technique used for surface digitization, presents several advantages over other optical techniques such as laser triangulation [12]. Among others, the ability for the reconstruction of high-sloped surfaces stands out, as does its lower dependence on surface optical properties [13].

In the literature, one of the most widely investigated materials is Ti6Al4V, an alpha-beta titanium alloy with high corrosion resistance, applied where low density and corrosion resistance are critical factors, such as in the aerospace industry and in biomechanical applications. Recently, the interaction between lasers and this alloy has been investigated, with particular attention to superficial treatments such as texturing.

For example in [14], a Yb-fiber laser is deployed to generate a quasi-uniform distribution of laser-induced periodic surface structures on titanium alloy substrates for adhesive bonding. In [15], Ti6Al4V micro-dimple surfaces fabricated by a masked laser surface texturing technique within water were subjected to soft and hard contact laser shock peening. In [16], the texturing was investigated of Ti6Al4V, by applying a pulsed Nd:YAG laser, aimed at surface characterization by microscopy.

Although many works can be found in the literature on analyses of laser surface treatments of Ti6Al4V titanium alloy, it seems there is not a systematic study showing the effects of main process laser parameters on shape geometry and roughness of parts fabricated by laser ablation. The aim of this paper was to investigate the shape geometry and roughness of Ti6Al4V features fabricated by laser milling using a nanosecond Nd:YAG laser source. The impact of the laser processing parameters on machining outcomes was studied in order to determine the optimal processing conditions, i.e., reducing geometrical defects and improving surface quality. In particular, the influence of average power, frequency and scan speed were investigated. Moreover, the laser machined 3D shape Ti6Al4V parts were measured and analyzed using CH technology. The Optimet Conoscan 4000 was employed with a spatial resolution of 12 μm × 12 μm and of 2.5 μm on the z axis.

## 2. Materials and Methods

### 2.1. Laser Machine Setup and Materials

All experimental tests were carried out on a laser machine equipped with a nanosecond Nd:YAG laser source pumped by laser diodes in Q-switch mode, and a scanning head which used deflecting mirrors to move the laser beam over the material surface. The laser spot diameter was constant at 70 μm. Plates of the Ti6Al4V titanium alloy with a thickness of 2 mm were used.

The laser system consisted of a nanosecond Nd:YAG laser source pumped by laser diodes, in Q-switch mode. The laser was characterized by a wavelength of 1064 nm and an average maximum power of 20 W. The pulse width was of 200 ns.

**Figure 1:**
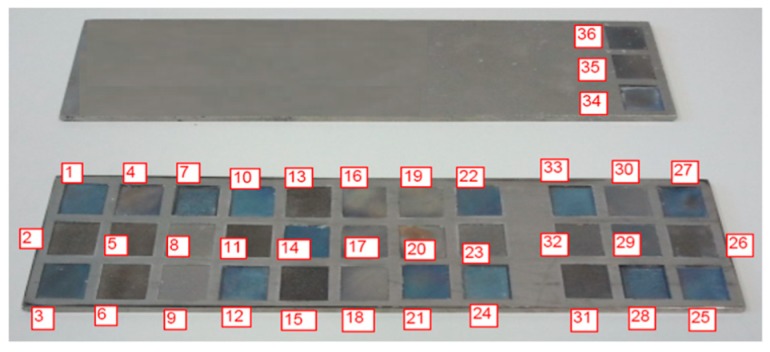
Built samples.

**Figure 2:**
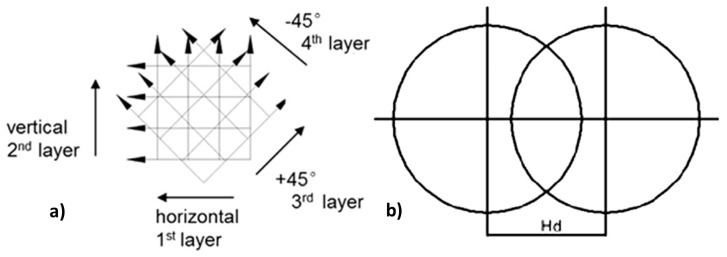
(**a**) Schematic representation of the employed scanning strategy; (**b**) Hatch distance between two consecutive laser pulses.

Square multilayer samples with dimension of 10 mm × 10 mm were manufactured (Figure 1). A Ti6Al4V plate was used as work piece material test. The investigation was conducted using a full factorial plan of experiments characterized by 3 factors (average laser power (P), scanning speed (v), frequency (F_p_)), with 3 levels for scanning speed (v), 3 levels for average laser power (P) and 4 levels for Frequency (F_p_), for a total of 36 combinations of parameters (Table 1). The scanning strategy used to remove the single manufactured layer was characterized by a mix hatching mode consisting of x and z axis parallel scanning vectors and ±45° tilted scanning vectors (Figure 2a). Every square chosen for tests was marked 100 times.

The hatch distance (Figure 2b) indicates the distance between two consecutive laser pulses. This factor has not been considered because it is related to scanning speed (v) and frequency (F_p_) by the Equation (1):

(1) $$\;H_{d}=\frac{v}{F_{p}}$$

The degree of overlap (O) of individual laser pulses can be calculated by the Equation (2), which considers also the spot diameter (D) [7]:

(2) $$O\%=\left[1-\frac{v}{F_{p}\times D}\right]\%$$

### 2.2. Dimensional Measurements

Dimensional measurements were performed with the 3D digitizing system Optimet Conoscan 4000. This combines a non-contact, single-point measuring sensor (Optimet ConoProbe Mark 3, equipped with a 50 HD mm lens, with claimed precision equal to 2.5 μm on the z axis and field of view equal to 2 mm) based on Conoscopic Holography (CH) technology with x-y axes repeatability (3σ) equal to 0.5 μm.

Measurements were obtained by setting a laser power approximately equal to 0.71 mW and a charge-coupled device (CCD) frequency power to 1000 Hz.

The choice of CH technology was mainly due to its capability to combine co-linearity with bending optics to measure deep, narrow slots, grooves and blind-holes. It can also be used simultaneously through the same focusing lens of welding and cutting lasers and machine vision cameras. This kind of sensor measures variable surfaces ranging from highly reflective, partially translucid, diffusive, to roughly textured surfaces with no need for painting or resurfacing. The 3D scan result is a dense point cloud with a very high amount of information regarding the dimension of the parts, where contact digitizer cannot touch the vertical shape of geometry, and conventional optical digitizing systems suffer problems related to undercuts and non-accessible areas.

**Figure 3:**
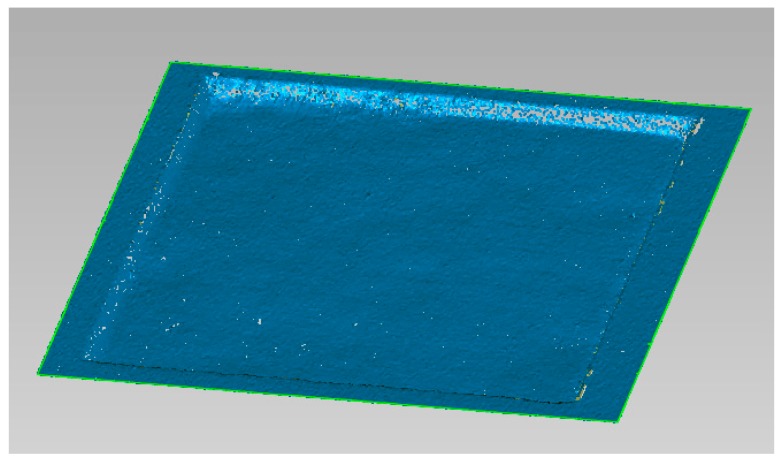
Example of point cloud.

Most of the work in literature presents experimental tests in which the dimensions of the part are obtained by cutting the specimens into three or more parts to obtain the cross-sectional profiles, and then measuring the depth and the width by digital images processed using optical microscopes. The use of the CH technology allows both the use of a non-destructive methodology, and the study of every micro dimension of the part. Figure 3 shows an example of benchmark acquisition.

**Figure 4:**
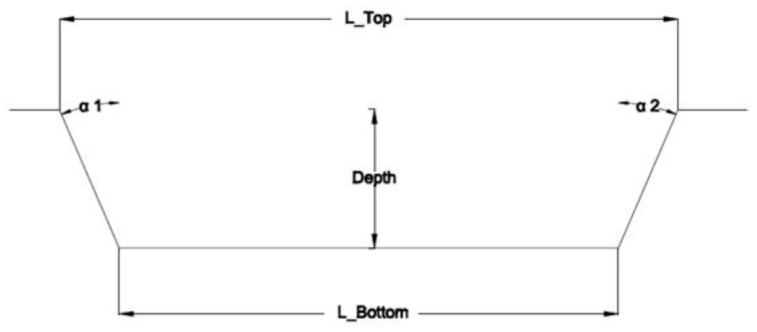
The five measured geometrical entities.

The five geometrical entities considered for each sample are shown in Figure 4 and consist of Top Width (L_Top), Bottom Width (L_Bottom), Depth, and two wall shape angles α1 and α2.

**Figure 5:**
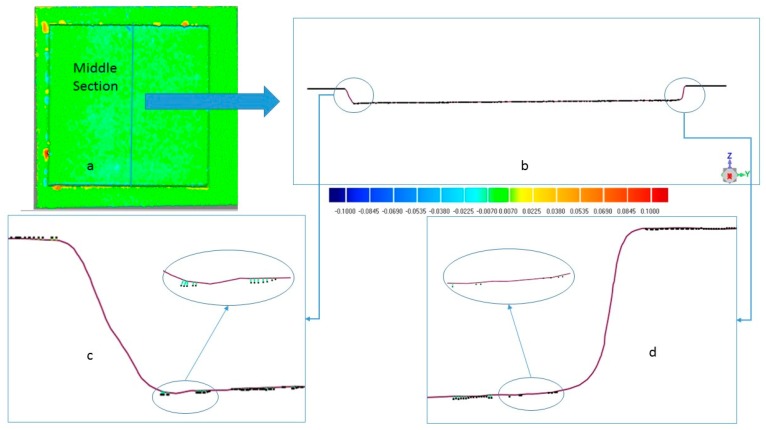
Comparison CH- optical profilometer for benchmark number 1. (**a**) Superimposition between CH and Taylor Hobson CCI MP-HS profilometer 3D models, (**b**) Middle Section, (**c**) Zoom of left angle (in violet the profile obtained from CH and in black the profile obtained from the profilometer), (**d**) Zoom of the right angle.

The use of CH has been validated, when possible, by the optical profilometer Taylor Hobson CCI MP-HS, equipped 10x objective with a displacement resolution of 0.01 nm on z axis and a scan range up to 2.2 mm without stitching. The validation was accomplished by comparing the CH point cloud with the profilometer one, after an Iterative Closest Point Procedure (ICP), with the commercial software Geomagic Control. Comparison was possible only in a limited number of samples due to the very different surface conditions between them. The optical profilometer is very sensitive to surface aspect, since it is easily affected by the angle of the features on the surface. When high roughness characterizes the surface, micro-features with high angles (micro-holes, sputters, etc.) significantly increase the difficulties of the measurement task. An example of a comparison is shown in Figure 5. Data obtained from the 3D comparisons are reported in Figure 5a, and have been retrieved using the commercial software Geomagic Control after a best-fit alignment between the interferometric scan data, identified as the reference, and photogrammetric scan data, identified as the system under test. After the manual identification of three points, the ICP algorithm [17] finds the nearest point of the Test for each point of the reference and computes the Euclidean distance. Each point of the test is associated with a distance, and the distances are clustered into colored intervals, according to the legend in the middle of Figure 5.

Apart from some defect areas, the predominant green color indicates a distance included into the interval [−7 µm, 7 µm], demonstrating a satisfactory level of accuracy of the CH technology. In Figure 5b, the middle section is shown where the continuous line indicates the section retrieved by CH; the black dots are the points retrieved by the profilometer. In Figure 5c,d the poor information retrieved by the optical profilometer on plane areas and the total absence of information on side walls may be seen.

**Figure 6:**
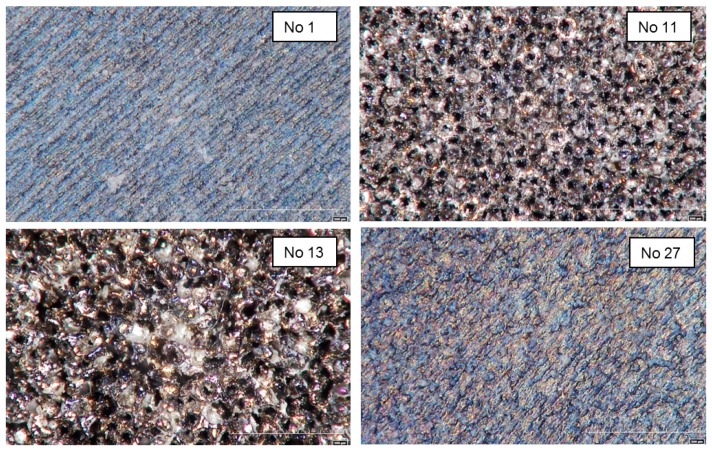
Microscope Images of several laser milled surfaces.

In Figure 6, some examples of milled surfaces are shown. Among these samples, only No. 1 was measurable with the optical profilometer.

Roughness tests were performed on all samples along the horizontal and vertical directions, as illustrated in Figure 2, in order to determine average values on the xy plane for all combinations of process parameters. The roughness Ram index was measured for roughness using the Taylor-Hobson Surtronic 25 instrument, and values of Ram were collected.

## 3. Results

### 3.1. Measurement Results

An error index (ER%) was introduced, according to Equation (3), to compare the single measured dimension (X_m_) with the single CAD dimension (X_CAD_).

(3) $$\mathrm{ER}\%=\frac{X_{m}-X_{\mathrm{CAD}}}{X_{\mathrm{CAD}}}\times 100$$

**Figure 7:**
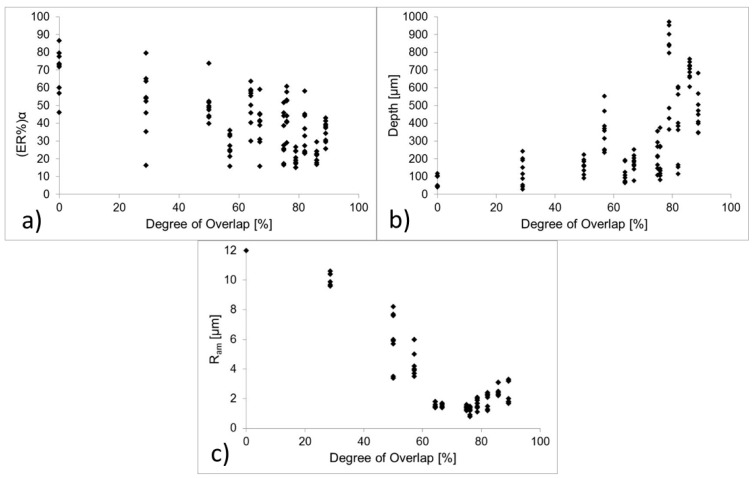
Tendency of (ER%)α (**a**) of Average Depth (**b**) and Roughness Ram (**c**) with the degree of overlap.

The analysis of results (Figure 7) showed that the average error on L_top was 0.587%, with maximum values of 2.9%. On the other hand, results for L_bottom showed an average error of 3.05% with maximum values of 6.77%. Results for wall angles α1 and α2 revealed an average error of 39.8% and 38.1%. An average value of wall shape angle (ER%)α was also calculated as the medium between the error on α1 and α2. This error ranges between a minimum value of 15% and a maximum of 86.4%. In this case, the error reaches very high percentages, as also confirmed by the literature [9].

### 3.2. Effect of Degree of Overlap on (ER%)α, Depth and Ram

Figure 7 shows the tendency of (ER%)α, of Average Depth and Ram with the degree of overlap. (ER%)α slowly decreases with the degree of overlapping, reaching a minimum value of about 15% for O% of about 80%. Average depth increases with the degree of Overlap, reaching a maximum value near to 1 mm for O% close to 80%. The highest values for Ram can be observed for lower O%. On the other hand, the smallest values for Ram are of 2 µm, corresponding to a degree of overlap between 64% and 79%. The tendency of Ram with O% are in line with previous studies of Campanelli et al. [4], that obtained a minimum value of Ram of 2.6 µm.

## 4. Discussion

In order to evaluate the influence of the considered process parameters on the error index calculated for single considered geometrical entities and on roughness, an Analysis of Variance (ANOVA) was performed [18]. The evaluated response results were ER L_Top, ER L_Bottom, (ER%)α, Depth and Ram.

The General Linear Model was used to perform ANOVA. Factors for this model are discrete variables; therefore, ANOVA examines whether the variance of the factor is zero. The p-value determines whether the effect for that term is significant. If the effect of a discrete factor is significant, then the variance of the factor is not zero.

The probability value α represents the factor coefficient; the smaller the value, the more significantly it represents the factor. In the present analysis, a threshold value of 0.05 was chosen. If the p-value is greater than the chosen level, the null hypothesis is accepted and the coefficient is considered not to be significant. Analysis of variance results are summarized in Table 2. The symbol (S) refers to the influence of each part parameter on the measured geometrical entities; the symbol (NS) means that the considered process parameter does not affect the result.

(ER%)α, Depth, and Ram result are affected by the variation of all the considered process parameters, while (ER% L_Top) is influenced only by (F_p_) and (ER% L_bottom) by both (v) and (F_p_).

**Figure 8:**
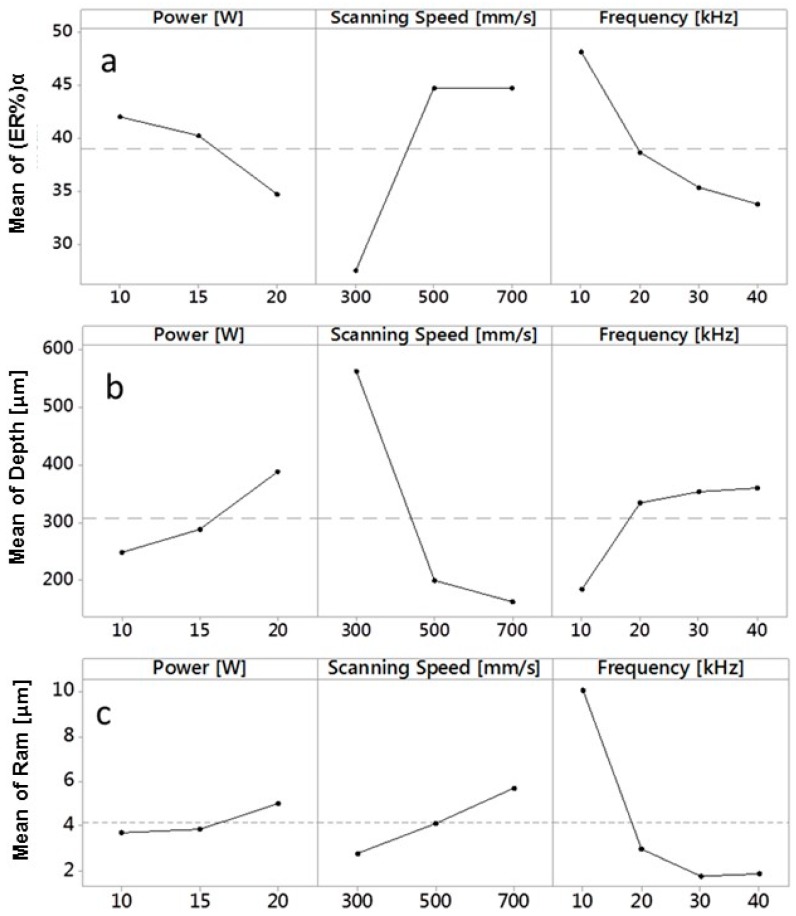
(**a**) Main effects plot for (ER%)α vs. Average Laser Power, Scanning Speed and Frequency; (**b**) Main effects plot for Depth vs. Average Laser Power, Scanning Speed and Frequency; (**c**) Main effects plot for Ram vs. Average Laser Power, Scanning Speed and Frequency.

Figure 8a–c show main effects plot for (ER%)α, Depth and Ram vs. the three input parameters Average Laser Power, Scanning Speed and Frequency. Some observations can be made:

- The slope of the curves confirms results of ANOVA on significance of input process parameters on analyzed results;
- The lowest value of (ER%)α can be obtained setting P = 20 W, v = 300 mm/min, F_p_ = 40 kHz.
- The lowest value of Ram can be obtained for P = 10 W, v = 300 mm/min and F_p_ = 30 kHz;
- A peak of Depth is present for P = 20 W, v = 300 mm/min, F_p_ = 40 kHz.

Moreover, it is possible to compare the relative magnitude of the factor effects, comparing the slopes of the lines on the plots. Plots for Ram show that by setting the Average Laser Power to within a range of 10–15 W, average roughness does not change significantly; the same consideration can be made for the variation of Frequency in the range 30–40 kHz.

Values of process parameters that minimize wall shape angle are the same that maximize Depth. This is due to the same definition of the wall shape angle, as assessed by Darwish et al. [6]. The wall shape angle depends on the difference of the channel’s width at top and bottom, in direct proportion, and two times the depth of channel, in inverse proportion. So, it can be said that wall shape angle is mainly controlled by Depth.

**Figure 9:**
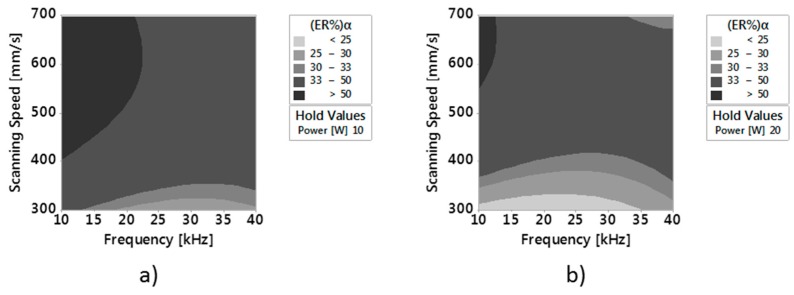
(**a**) Contour Plot of (ER%)α versus v and F_p_ at constant Power of respectively 10 W and (**b**) 20 W.

**Figure 10:**
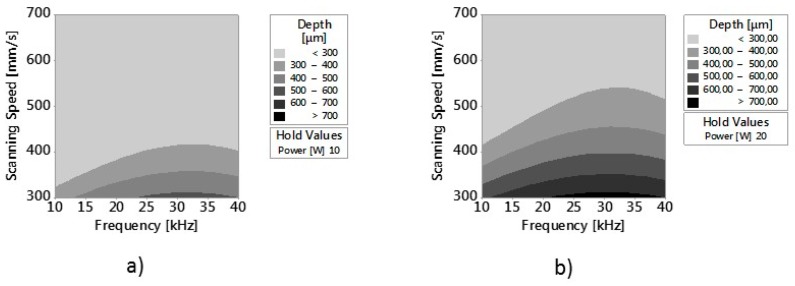
(**a**) Contour Plot of Depth versus v and F_p_ at constant P of respectively 10 W and (**b**) 20 W.

After ANOVA, the response surface methodology (RSM) was used for modeling and analyzing the response of the single outputs with the considered factors. The contour plot methodology was used to visualize the response surface. Figure 9 and Figure 10 show contour plots of (ER%)α and Depth versus v and F_p_ at constant Average Laser Power of respectively 10 W and 20 W. Since the purpose of this work is also to analyze the reduction of the error, and at the same time to get the best surface quality, laser power was set to these two levels (the minimum and the maximum of the considered range). Indeed, results of the previous analysis on main effects showed that the minimum values of Ram and (ER%)α are obtained respectively for P = 10 W and P = 20 W.

The contour Plots of (ER%)α for the two different P values confirm that values of (ER%)α lower than 25% can be obtained for P = 20 W, F_p_ between 10 kHz and 35 kHz and v equal or slightly higher than 300 mm/min.

**Figure 11:**
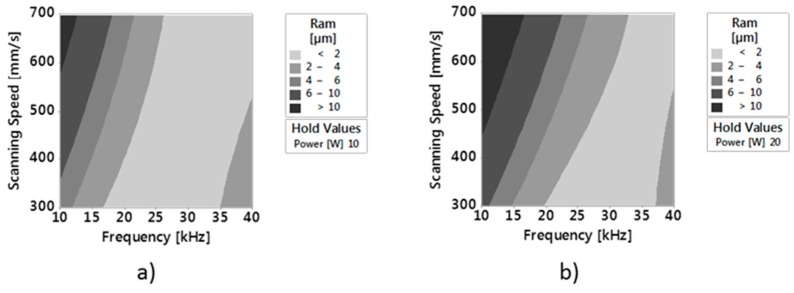
(**a**) Contour Plot of Ram versus v and F_p_ at constant P of respectively 10 W and (**b**) 20 W.

Comparing these values to those of Ram (Figure 11), it can be observed that for the same average laser power and scanning speed values, values of Ram lower than 2 μm can be found in a frequency range of 20–37 kHz.

**Figure 12:**
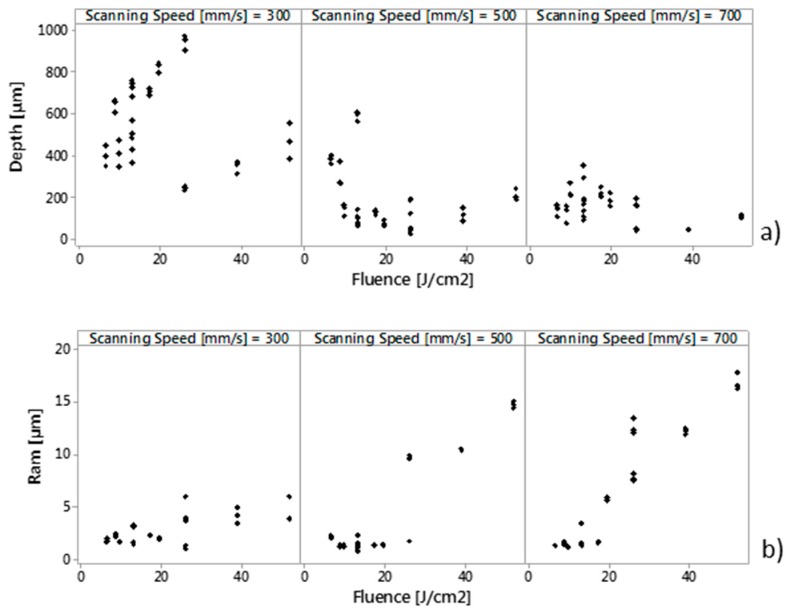
Scatterplot of Depth (μm) (**a**) and Ram (**b**) vs. Fluence (J/cm^2^) with panel variable scanning speed.

Results for roughness can be related to the degree of overlap, as demonstrated in Figure 7, in which can be detected a range of O% between 64% and 79% in which Ram shows values lower than 2 μm. However, Ram it is also affected by the optical energy delivered per unit area (fluence). Figure 12b shows, the scatterplot of Ram vs. Fluence (J/cm^2^) with panel variable scanning speed. At lower values of fluence (<20 J/cm^2^), roughness is very low and it seems not to be much affected by energy; at higher values of fluence (>20 J/cm^2^) roughness increases slowly for v = 300 mm/s e faster at v = 500 and 700 mm/s. Similar results were found by Campanelli et al. [4] on laser ablation of 5754 aluminum alloy.

Figure 10a,b confirm that maximum values of Depth are found per the minimum scanning speed v = 300 mm/s. This result is validated by Figure 12b, which shows scatterplot of Depth (μm) vs. Fluence (J/cm^2^) with panel variable scanning speed. For the highest scanning speed (v = 700 mm/s) depth seems to have only a slight variation with laser fluence. This is due to the lower interaction time between the laser beam and the machined surface [4]. For lower values of speeds (300 and 400 mm/s), depth increases quickly for lower values, and slowly for higher values of fluence.

ER%α shows a slight reduction with the degree of Overlap, but there is large data dispersion; thus, the variation of ER%α cannot be attributed entirely to O%, but is also due to lower machining depths, as also assessed by Darwish et al. [6].

In conclusion, the analyzed results show that by setting appropriate process parameter values, it is possible to reduce the error on vertical walls, and at the same time, increase the surface quality.

## 5. Conclusions

In this paper, an experimental work to investigate the shape and the dimensions of Ti6Al4V micro-parts fabricated by nanosecond laser milling was performed. Dimensional measurements were performed with the 3D digitizing system Optimet MiniConoscan 4000, based on CH technology with claimed precision equal to 2.5 μm on the z axis, field of view equal to 2 mm. CH technology revealed itself to be useful and better at retrieving the side walls compared to other optical technologies, such confocal microscopy and interferometric profilometry. The effect of three main process parameters (average laser power, scanning speed and frequency) on the error index, calculated for the single considered geometrical entities, and on surface roughness, was studied. Moreover, the Response Surface Methodology, applied for modeling the response of the single outputs with the considered factors, showed that:

- Wall shape angles can be controlled setting appropriate values of process parameters.
- Values of roughness lower than 2 µm can be obtained setting proper values of power, frequency and scanning speed
- Values of roughness lower than 2 µm and of (ER%)α lower than 25% can be obtained with an average power of 20 W, Frequency between 20 kHz and 35 kHz and scanning speeds equal to or slightly higher than 300 mm/min.

## Figures and Tables

**Table 1:** Experimental plane.

Sample	Average Laser Power (W)	Scanning Speed (mm/s)	Frequency (kHz)	Degree of Overlap (%)
1	10	500	40	82
2	20	500	10	29
3	10	500	30	76
4	15	300	40	89
5	10	500	10	29
6	15	500	10	29
7	20	300	20	79
8	15	700	20	50
9	10	700	20	50
10	10	300	20	79
11	10	700	10	0
12	20	300	30	86
13	20	700	10	0
14	20	700	40	75
15	20	300	10	57
16	20	500	30	76
17	10	500	20	64
18	10	700	30	67
19	15	500	20	64
20	15	500	30	76
21	15	700	40	75
22	20	700	30	67
23	20	700	20	50
24	15	300	30	86
25	10	300	30	86
26	15	700	10	0
27	20	300	40	89
28	15	300	20	79
29	15	700	30	67
30	10	700	40	75
31	15	300	10	57
32	10	300	10	57
33	20	500	40	82
34	10	300	40	89
35	15	500	40	82
36	20	500	20	64

**Table 2:** Significance of factors vs. geometrical entities.

Geometrical Entities	Average Laser Power (W)	Scanning Speed (mm/s)	Frequency (kHz)
ER L Top (ER% L_Top)	NS	NS	S
ER L Bottom (ER% L_bottom)	NS	S	S
(ER%)α	S	S	S
Depth	S	S	S
Ram	S	S	S

## Nomenclature

P	average laser power
F_p_	frequency
v	scanning speed
Hd	hatch distance
O	degree of overlap
D	laser spot diameter
ER	error index
X_m_	single measured dimension
X_CAD_	single CAD dimension
Ram	arithmetic average roughness
α	probability error
L_Top	channel top Width,
L_Bottom	channel bottom width
α1, α2	channel side angles
